# Supplementary material for: Silicon as a ubiquitous contaminant in graphene derivatives with significant impact on device performance
Source: Nat Commun. 2018 Nov 29;9:5070. doi: 10.1038/s41467-018-07396-3 (PMC6265250; doi:10.1038/s41467-018-07396-3)
Supplement: Supplementary file 1 — Supplementary Information [file 41467_2018_7396_MOESM1_ESM.pdf]

Supporting Information

Rouhollah Jalili[1]*, Dorna Esrafilzadeh[2]*, Seyed Hamed Aboutalebi[3,4,5], Ylias M. Sabri[6], Ahmad E. Kandjani[6], Suresh K. Bhargava[6], Enrico Della Gaspera[1], Thomas R. Gengenbach[7], Ashley Walker[8], Yunfeng Chao[8], Caiyun Wang[8], Hossein Alimadadi[9,10], David R. G. Mitchell[11], David L. Officer[8], Douglas R. Macfarlane[12], Gordon G. Wallace[8]

[1]School of Science, RMIT University, Melbourne, VIC, 3001, Australia.
[2]School of Engineering, RMIT University, Melbourne, VIC, 3001, Australia.
[3]Institute for Superconducting and Electronic Materials, Australian Institute for Innovative Materials, University of Wollongong, Wollongong, NSW, 2522, Australia.
[4] Pasargad Institute of Advanced Innovative Solutions, Tehran
[5]Condensed Matter National Laboratory, Institute for Research in Fundamental Sciences, 19395-5531, Tehran.
[6]Centre for Advanced Materials and Industrial Chemistry (CAMIC), School of Science, RMIT University, Melbourne, VIC, 3001, Australia.
[7]Manufacturing, Commonwealth Scientific and Industrial Research Organisation, Clayton, VIC, 3168, Australia
[8]ARC Centre of Excellence for Electromaterials Science, University of Wollongong, Wollongong, NSW, 2522, Australia.
[9]DTU Danchip/Cen, Technical University of Denmark, Center for Electron Nanoscopy, Fysikvej, Building 307, 2800 Kgs. Lyngby, Denmark.
[10] Danish Technological Institute, Kongsvang Alle´ 29, 8000 Aarhus C, Denmark.
[11]Electron Microscopy Centre, Australian Institute for Innovative Materials, University of Wollongong, Wollongong, NSW, 2522, Australia.
[12]ARC Centre of Excellence for Electromaterials Science, Monash University, Clayton, VIC, 3800, Australia.

*Correspondence to: ali.jalili@rmit.edu.au, Dorna.esrafilzadeh@rmit.edu.au

Supplementary Discussion:

Further discussion on the STEM techniques and analysis. Atomic structural imaging in the STEM works well for bulk crystalline materials. However, for this class of material there are a number of insurmountable challenges. The silicon-containing impurity is amorphous. Clusters of the silicon-containing species are diffuse and there is clearly no defined structural relationship with the underlying graphene. This is in contrast to metal atom clusters (eg Fe). These often develop platelets with a loose, albeit emergent, ordering. The biggest problem is exemplified in Figure S6, which shows how the specimen is damaged by the electron beam. Aside from holes opening up in the graphene, the adatoms/clusters become highly mobile under the intense electron flux (we use the lowest practical probe current possible 30pA), and this imposes an upper limit on the useful magnification/imaging time at which such features can be imaged/analysed without modifying them. This was despite using the lowest operating voltage for our STEM (80kV) to minimise knock-on damage.

Selected area diffraction is a TEM-based technique. It relies on the use of an area-defining aperture to select a region from which to form a diffraction pattern. Due to the inherent

uncertainty in the selection of a region for diffraction, the smallest usable diameter of a selected area aperture is around 200nm. Given that the clusters we are describing are atomically dispersed and very small in diameter (1-2 nm), and given that they represent a small fraction of the film – being monolayers in thickness), such features could not be expected to produce any measureable scattering. The Si-rich regions are amorphous, and as such any scattered intensity would be diffuse further exacerbating the problem. At this scale, a more useful approach is the atomic resolution HAADF imaging (STEM-based) method we have used. This allows the atomic locations/single atoms to be directly visualised, and crystalline materials to be identified by virtue of their periodic structure. Note, an aperiodic structure in an image does not necessarily indicate an amorphous material, since it may simply not be oriented at a strong Bragg condition.

Given that the Si-rich regions we have observed on many dozens of different specimens are all aperiodic it is safe to assume that this phase is amorphous (as one might expect for Si of this nature). One advantage that the HAADF approach is that it does permit sub-areas of an image to be processed using Fourier methods to create a diffractogram. This highlights the periodic structures in that region, providing information which can be analogous to that in a selected area diffraction pattern, albeit from regions as small as several nm in diameter. Clearly, it is possible to process the images we have included in the paper to extract such diffractograms from sub-areas of interest. However, the usual motivation for so doing to is to highlight periodic information, often the orientation relationship between two phases. In the case of the Si phase this would not offer any advantage, since the image sub-areas containing Si-rich regions show no periodic information, and therefore, neither would the diffractograms.

Aberration-corrected STEM is possible by virtue of forming an electron probe with a diameter of around 0.1nm. In this work, we were constrained to use an 80kV accelerating voltage, rather than the more typical 200kV beam energy (the latter providing higher spatial resolution). The low voltage helped reduce the knock-on beam damage of the material. Obtaining atomic resolution images at 80kV requires the use of the smallest imaging probe on the instrument. This very small size is achieved at the expense of electron current in the probe, which proportionately reduces the x-ray yield. One must also remember that typical STEM specimens may be 5 to 100nm in thickness, and so the excited volume for x-ray generation is (relatively) large. Here, we are dealing with a material which has sub-nm thickness, resulting in a vanishingly small x-ray yield. For analytical (EDS) work, probes with an order of magnitude more current are typically used – although they are much larger

(ca 0.2nm), and typically do not provide atomic resolution. So, the low voltage work necessary to avoid damaging this class of 2D materials necessitates the choice of a probe which enables either atomic resolution or 2D x-ray mapping – but not both (at 200kV it is possible to have both). For this reason, we were constrained to carry out localised point analysis with a low current probe by scanning impurity sub-areas and integrating the EDS spectra over periods of up to 240 s (for a single point). Even if we were prepared to sacrifice resolution and use a larger current probe, another problem is that intense beams mobilise surface atoms and probed clusters simply disintegrate as the atoms/molecules diffuse away from the beam (please see Figure S6). In summary, 2D x-ray mapping of this class of material at 80kV at this scale is not possible.

EELS has been used to provide structural and electronic information on graphene-based materials. However, such studies are beyond the scope of the current work where the focus is not on the graphene oxide per se, but rather on the nature of the impurities in graphene oxide, their composition and distribution and their influence on the performance of graphene-oxide-based devices. There are several motivations for doing EELS, which include thickness measurement, compositional analysis and structural/electronic characterization. Since our focus was on understanding the composition of the impurity regions we selected EDS analysis as the tool of choice. Not only is it simpler and less prone to artefacts than EELS, but it provides information on the entire periodic table from B to U, whereas EELS is best suited to light elements due to their higher scattering cross-sections.

Further discussion on the humidity sensors. Detection of relative humidity (RH) is extremely important in monitoring the environment, human/animal respiration, food quality, indoor air quality, chemical industry processing and trace moisture in high-purity gases in the semiconductor industry [1]. The ideal RH sensor would have the following properties: low limit of detection, accuracy over the whole-range from trace moisture measurements to high humidity levels, ease of device manufacturing and low cost of device fabrication, ultra-high selectivity in the presence of prevalent gases in practical environments, high stability and recovery rate and short recovery time. A GO based device has shown great promise in this respect [2,3]. However, it still falls short of the low limit of detection of 2D MXenes which have the lowest limit of detection of 0.8% RH [4]. The detection range and moisture uptake reported so far, although promising, represents an incremental improvement over existing technologies and does not yet offer a practical implementation of 2D materials for this

application. Various types of humidity sensors have been reported over the years. However, the development of humidity sensors with a wide detection range, high sensitivity and short recovery times is still a challenge. To achieve these goals, considerable attention has been directed toward the development of humidity sensitive materials with recent use of graphene[5], graphene oxide (GO)[2,3,6-8], GO composites[9-11], black phosphorus (BP)[12], gold[13], MXenes[14] and organic molecules[1,15,16] on different sensing transducers. These can detect humidity due to their high surface-to-volume ratio, low noise, high moisture affinity and low sensitivity of electronic properties to variations in environment. Although some of the materials/device combinations developed so far have shown good performance in some aspects of RH sensing, there have been trade-offs in other aspects such as sensitivity, repeatability, response time, concentration detection range and/or selectivity performances.

In order to investigate the effect of the purity of our materials on their sensing response, the synthesized materials were deposited on the Ti QCMs and exposed to a set of various humidity concentrations at 27 ºC, with humidity levels varied from low to high. The limit of detection was calculated based on the standard deviation of the sensor noise. This involved calculating the standard deviation of the sensor noise over a 15-minute period (i.e. the exposure time) of three different blank profiles. We then converted this back to humidity concentration using the calibration curve to convert the Δf signal to humidity content[17]. This was performed by considering the fact that the sensors exhibited low drift[17]. This allows us fit a linear curve for the first lowest 5 humidity concentrations tested.

It was observed that all three modified QCMs produced extraordinary responses toward even the smallest humidity level indicating extremely high humidity uptake capacity, the extent of which has not been reported to date. The control Ti QCM (having no GO) had no response to humidity as expected, indicating that the QCM responses were due solely to the presence of GO. The QCM data shows that the surfaces have extremely high affinity toward humidity (average sensitivity of 66.5 Hz/%RH). To date, the highest reported sensitivity for QCM based humidity sensors was reported at 28.7 Hz/%RH using a copper metal-organic framework as the sensitive layer[18] (see comparisons in Table S1). The HR sensors developed here rely on the massive water absorption capacity of the hydrophilic functionalities on the surface of GO[19]. This is in contrast to systems which rely on capacitance or potentiometric methods used in other studies (table S1). All of the sensors developed here showed superior performance relative to the best GO-based HR sensors reported in the literature. Moreover, it appears that the water absorption capacity of GO is much higher than that of other active

materials used in the best performing QCM-based sensors such as gold, copper and MXenes. This is due to the fact that almost all of the hydrophilic functional groups of GO are present at the surface, since there is no bulk in 2D materials. Therefore, even the GO of lowest purity used in this study showed better performance compared to other reports in the literature. Moreover, the purity of the material surface becomes the key point to determine the final performance. Therefore, we could improve the performance even further by eliminating the silicon-based impurity.

Supplementary Table 1. Benchmarking the GO-based humidity sensor against some of the best reported humidity sensors.

No.	Material	Humidity Range (%RH)	LOD* (%RH)	Sensitivity (/%RH)	Sorption capacity	Selectivity (%)	Sensing technique	Ref
1	Au	12.5-90 @30°C	12.5* @30°C	-	-	64	QCM	13
2	BP	12.5-90 @30°C	10 @30°C	1.04 Hz/pg	-	83	QCM	13
3	Cu(II)-MOF	17.2-97.6 @ 25°C	17.2* @ 25°C	28.7 Hz	-	75	QCM	18
4	MXenes (Mg, K)	20-85 @ 27°C	0.8 @ 27°C	-	0.025% @	-	QCM	4
5	GO (2D)	15-95 @25°C	15* @25°C	52.86 pF	-	-	Capacitance	6
6	Si-NW	11.3-93 @25°C	11.3* @25°C	14.4 pF	-	-	Capacitance	20
7	GO multilayer	10-98 @25°C	10* @25°C	79.3 µV	-	-	Potentiometric	21
8	SnO_2–NW	30-85 @30°C	30* @30°C	19 nA	-	-	Resistive	22
9	MoS2 (2D)	0-35 @~27°C	5* @~27°C	10^5 Ω	-	-	FET	23
10	Pure GO	2.5-90 @27°C	0.006 @27°C	153 Hz	>30% (90%RH @ 27°C)	94	QCM	This study

* Lowest experimental concentration where low limit of detection (LOD) is not reported
# Selectivity was reported against the most extreme interfering scenario (reported response ratio)

The humidity uptake of the GO materials was achieved with a response time (calculated based on t_{90} standard [17]) of only 35 seconds (t_{90} value for clean GO). This response time is very fast when compared to other humidity sensor technologies [6], indicating that the sorption rate is high and making it highly suitable for humidity sensing applications. To demonstrate

the significance of such high capacity, a recent study [14] undertaking gravimetric experiments on one of the best humidity sensing materials (MXenes; Mg, K) showed a 0.025% humidity uptake (35 ng H_2O per 140 µg material) resulting in a high S/N ratio and lower limit of detection (LOD) of 0.8% RH. In comparison, the lower limit of detection (LOD) of the clean GO-based humidity sensor in this study (calculated by 3 standard deviations of the blank sample noise profile) [4] was as low as 0.006 %RH at 27ºC which is equivalent to 1.5 mg/m^3 (2 ppm) absolute humidity. Such a low LOD value for humidity sensors has never been reported before; the lowest LODs being an order of magnitude higher at >0.05 %RH [1,15]. Furthermore, following each humidity exposure event, the sensors recovered at >99.8%, the recovery being readily achieved by exposure to dry nitrogen, with recovery times (t_{90-r}) being less than 1 minute. Given that the sensors working range is shown to be from trace levels and up to >90% RH, the sensor can be used for any application requiring humidity sensing. Relative humidity sensors based on two-dimensional (2D) materials reported in literature generally do not span such a large detection range[3,6,11,17].

The sensors' selectivity performance toward humidity in the presence of other industrially relevant contaminant gases was also outstanding. The contaminant gases were chosen to represent some industrially relevant gases at high concentrations in order to simulate the harshest of industrial environments. One such example is the alumina industry's digestion stack [24]. The GO sensors developed in this study, were found to be highly selective towards humidity even in the presence of very high concentrations of contaminant gases. There is no data on this type of selectivity test reported in the literature previously, so comparisons cannot be made. The data here showed that the sensors were highly selective toward humidity in the presence of all gases tested here, including the presence of mercury vapor at high concentration (24 ppb).

The humidity sensor we have developed here using pure graphite showed outstanding selectivity and sensitivity and can be applied in practical environmental monitoring systems. Given the wide range of humidity levels it is able to detect, the sensor developed in this work could eliminate the need for employing multiple sensors to detect different ranges of humidity. Given that QCMs are mass-based devices and that physical interactions between the hydrophilic functionalities of GO and humidity provide the basis of the humidity sensing mechanism, the sensors were easily regenerated using an exposure to dry nitrogen, without any external energy input being required. Such a high performance graphenic material provides an enabling platform for trace moisture measurement in complex systems. This

unprecedented performance can be attributed to the much higher available hydrophilic surface area of the employed GO (99.9 % purity) for water absorption/desorption when the surface contamination is eliminated.

Further discussion on XPS: additional effects of etching on GO. As mentioned in the main text even under the very mild etching conditions used, the bombardment of the GO surface with Ar clusters caused a significant reduction of the GO. This effect has been reported previously.[25] It highlights a potential problem (and limitation) of the technique: the combined effects of ion bombardment, both direct (collision of Ar clusters with surface species) and indirect (surface heating) results in a change of surface chemical structure, in addition to simply removing surface material. In addition, heating of the GO would also enhance mobility of organosilicon contaminants and facilitate diffusion through the material. While not changing, the main conclusion derived from XPS depth profiling these experimental artefacts might increase the uncertainty associated with interpretation and quantification of the results.

Supporting Figures

Microscopy:

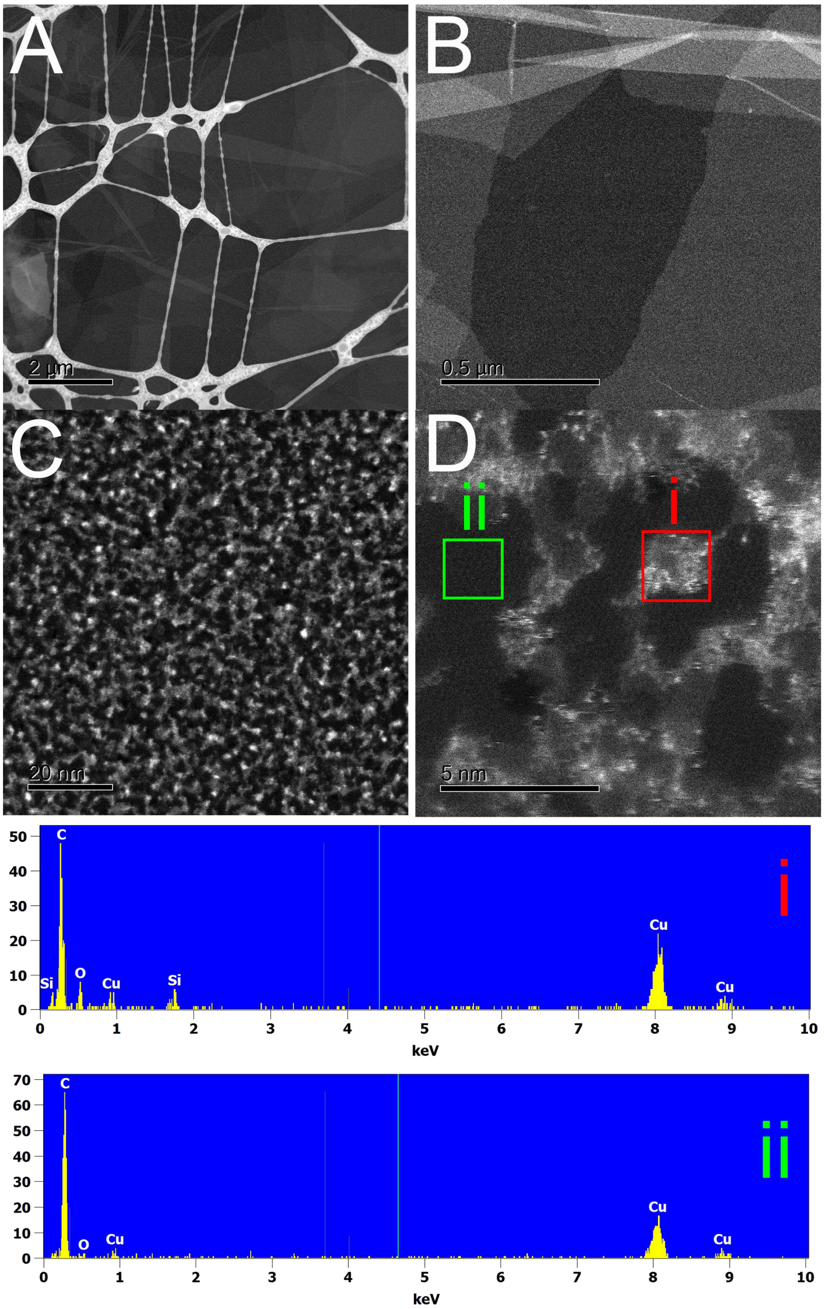

Supplementary Figure 1. HAADF images of GO from graphite (98% purity) at various magnifications. The green-boxed area in D (ii) shows a perfect graphitic lattice structure with only carbon peak presents at the EDS spectra. On the other hand, a silicon-based impurity was present in the red-boxed region (i) resulting in detectable Si and O peaks in the EDS spectrum. The Cu signal originates from the copper support grid. No calcium (Kα. 3.690 eV) was detected. This contaminant can be present in GO if not very pure water is used. Even using very pure graphite (99.9999%), occasional scattered atoms of Ca were detected, and these may have come from the water (discussed in Fig S6).

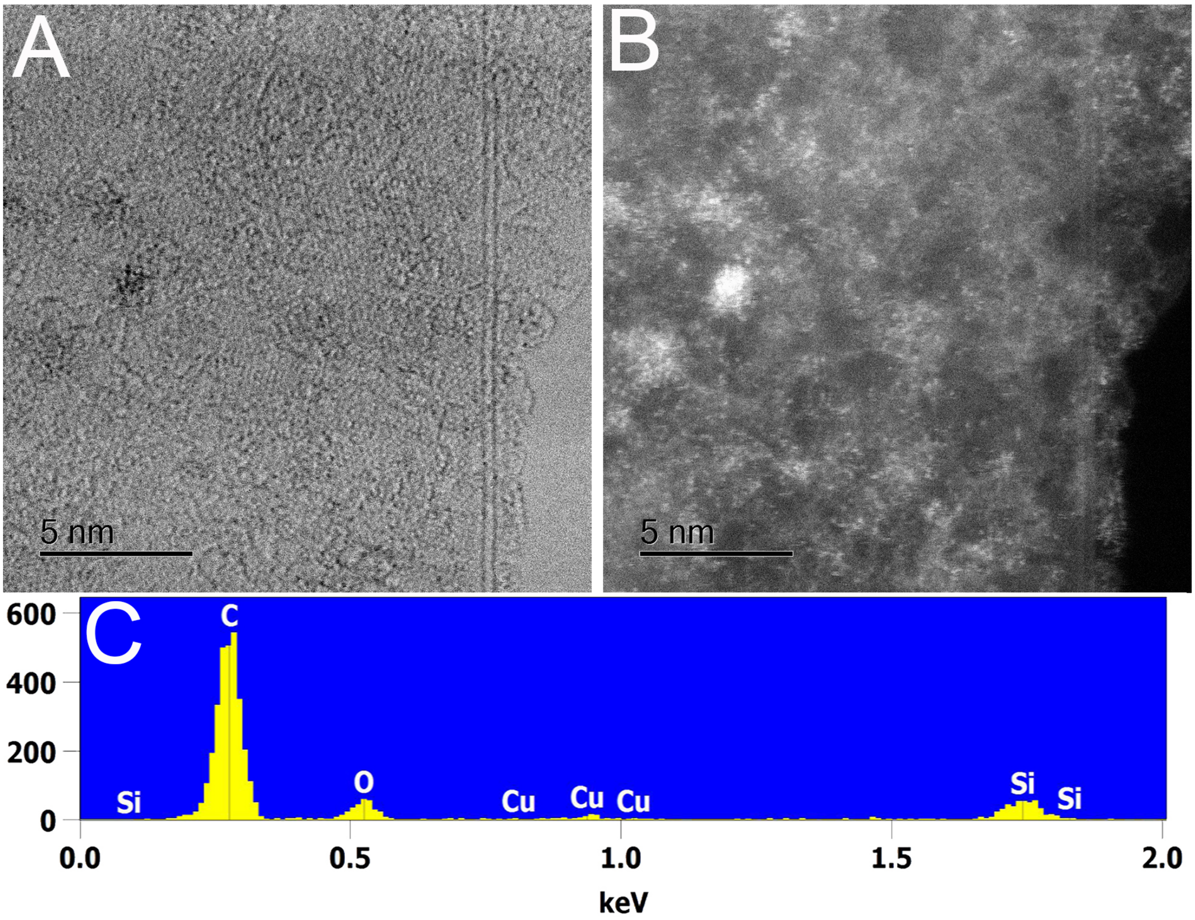

Supplementary Figure 2. A) Bright field and B) HAADF images of solvent exfoliated graphene from graphite (98% purity). C) The EDS spectra shows significant amount of silicon-based impurities.

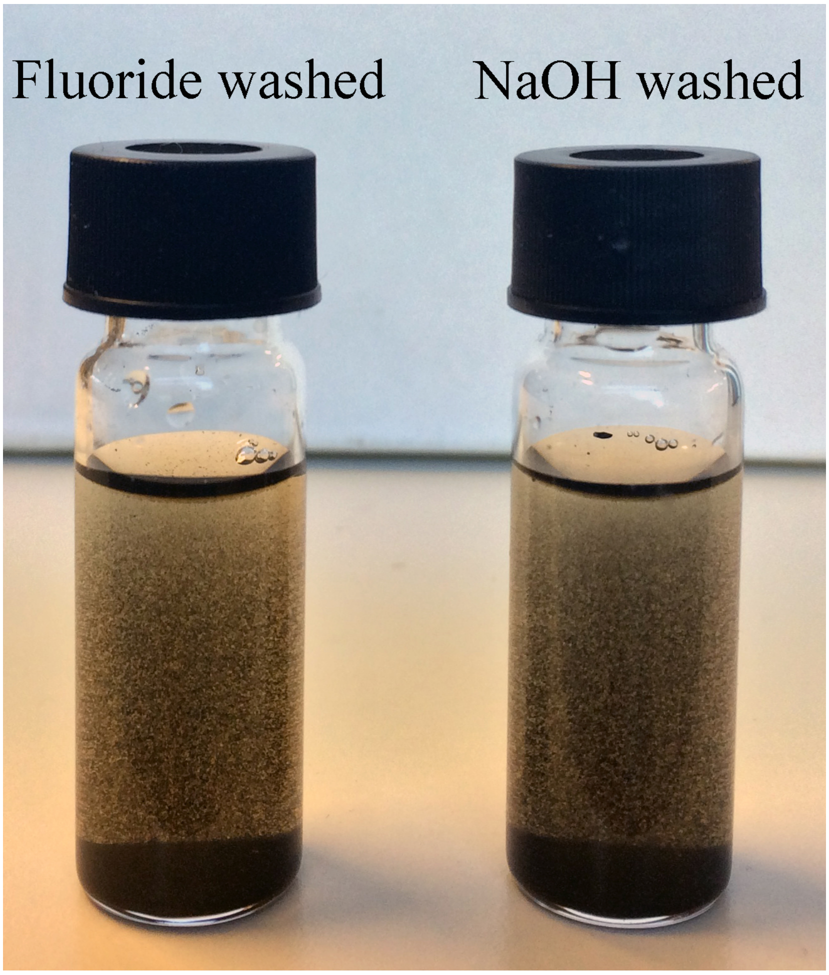

Supplementary Figure 3. Photograph of GO dispersions after extensively washing with Fluoride and NaOH. Purification with such a strong basic solution resulted in an irreversible agglomeration and restacking of GO sheets. Generally, increasing the ionic strength or decreasing the pH of GO suspensions results in loss of the surface charge and restacking of GO particles then occurs.

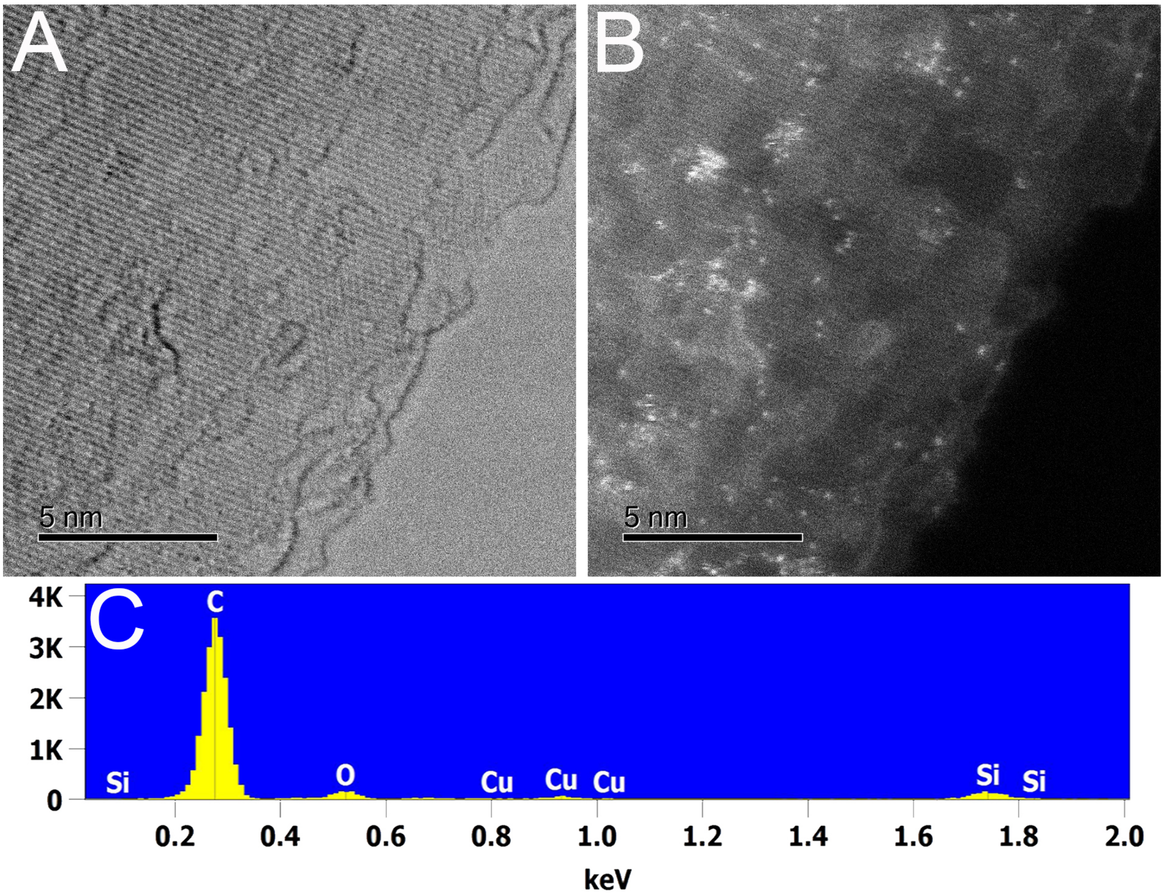

Supplementary Figure 4. A) Bright field and B) HAADF images of fluoride-washed GO made from low purity (98%) graphite. Restacking of the sheets is evidenced by the BF image. C) The EDS spectrum shows the persitence of silicon-based impurities. Apart from the additional cost of washing, the main limitation is agglomeration of graphene sheets following washing. GO, for example, is a colloidal dispersion that is very sensitive to any change in the charge and pH. Acid and base washing (HF, NH_4F and NaOH) result in irreversible agglomeration. Agglomeration also makes the washing process inefficient as contaminants get trapped between the stacked sheets.

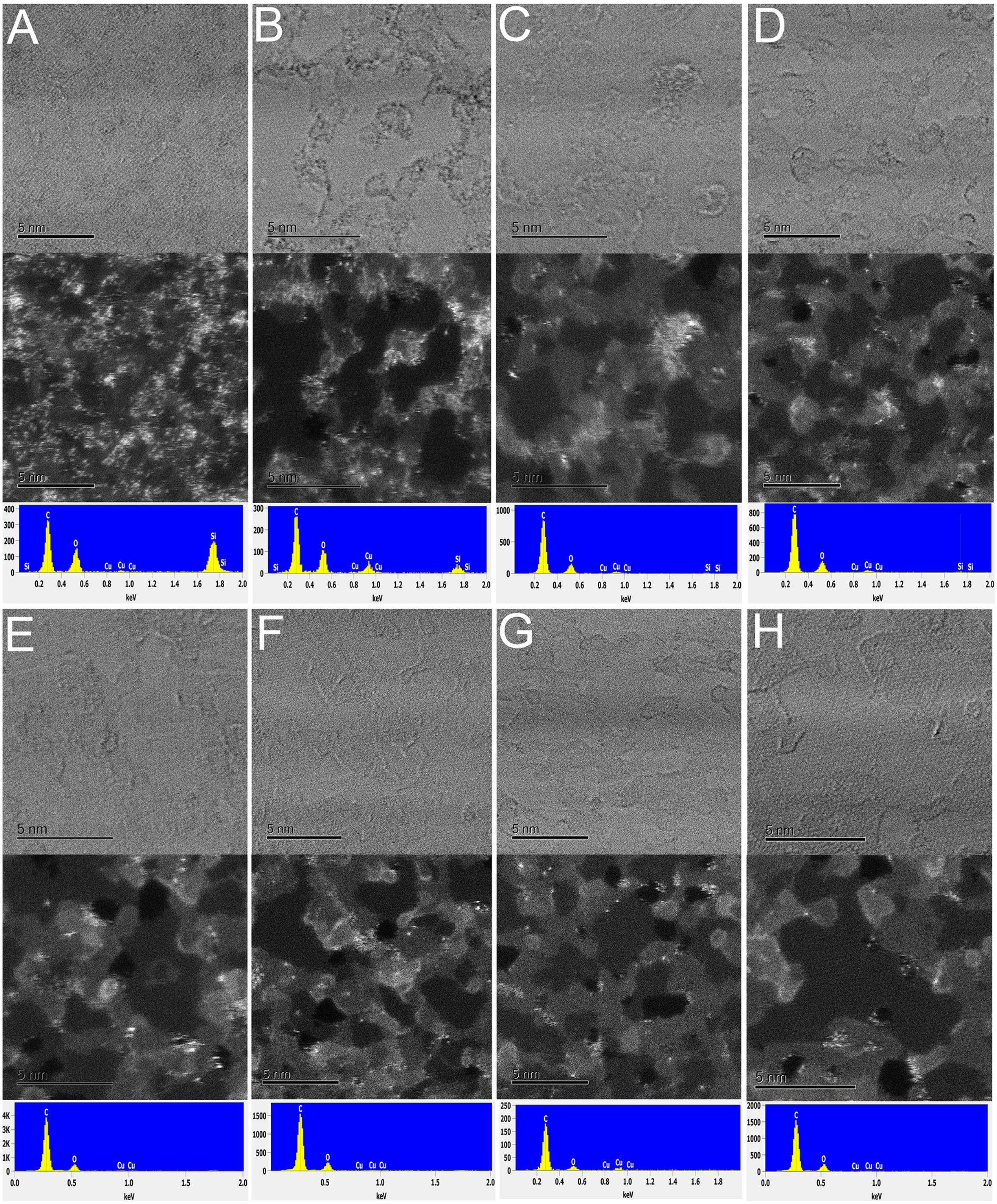

Supplementary Figure 5. Comparison of STEM images and EDS spectra of graphene oxides (GO) synthesised using graphite of various purities. Top BF, middle HAADF and bottom EDS spectrum. In contrast to conventional BF images in which silicon-rich contaminants are undetectable, the HAADF images identified the silicon-based impurities as bright spots/clusters. A) Commercial GO, B) natural graphite flake (98 %), C) natural graphite flake (99 %). D) natural graphite flake (99.8 %). E) natural graphite flake (99.9 %). F) natural graphite powder (99.9995 %). G) synthetic graphite powder (99.9995 %). H) (natural graphite powder of 99.9999 %). Si atoms appear as white dots in the HAADF images. The peaks at ~0.277, 0.525 and 1.739 keV in the EDS spectra are due to C, O and Si respectively. The peak at 0.930 keV is from the Cu support grid.

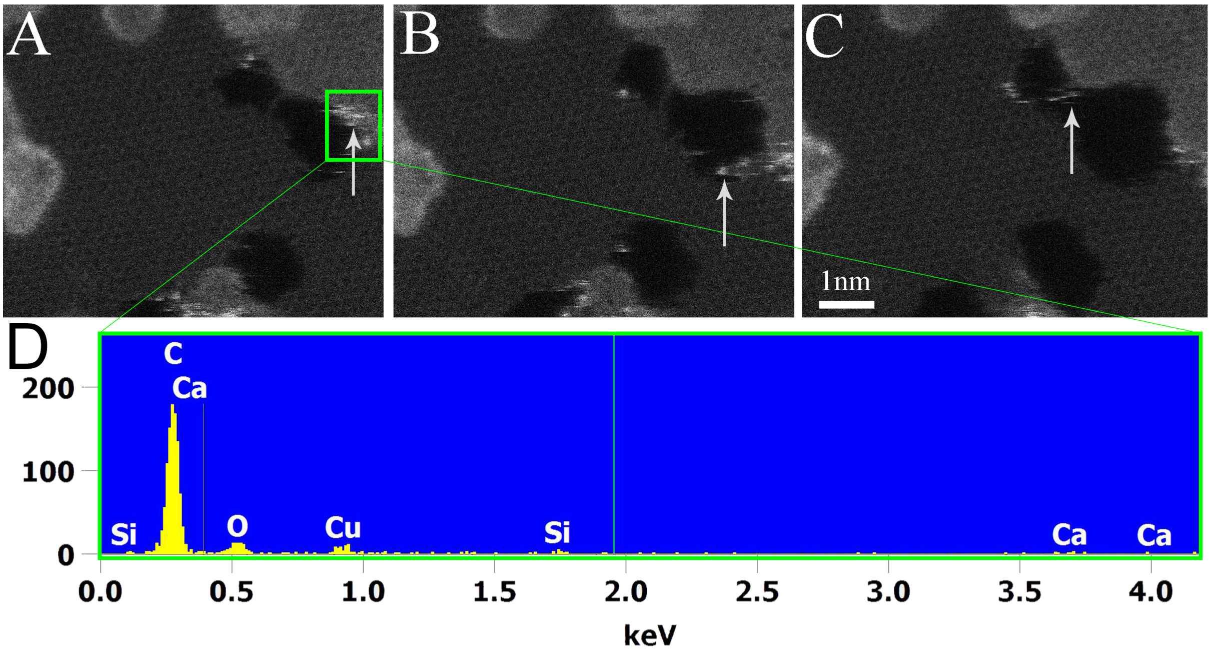

Supplementary Figure 6. A-C) Comparison of three consecutive HAADF images of GO made from high purity graphite (99.9999%), time lapse between successive frames is ~ 1 min. The bright spots marked with arrows in the images are atoms of contaminants that are mainly silicon-based and also calcium (possible from water). Under the exposure of the beam, these atoms move freely on the surface and open holes on the GO sheet. D) EDS spectrum of the boxed region in A showing trace amounts of Si and Ca.

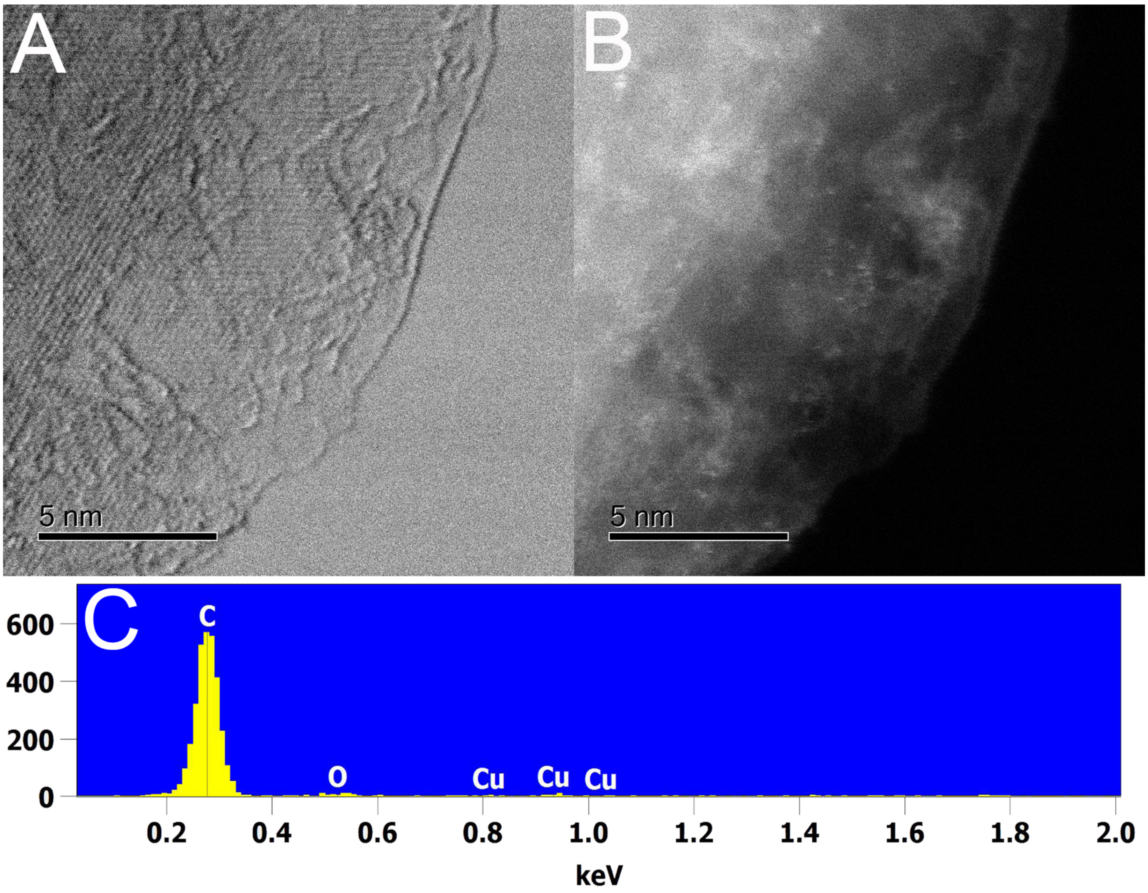

Supplementary Figure 7. A) Bright field and B) HAADF images of graphite (99.9% purity). C) The EDS spectrum shows only carbon peak and very little oxygen or silicon.

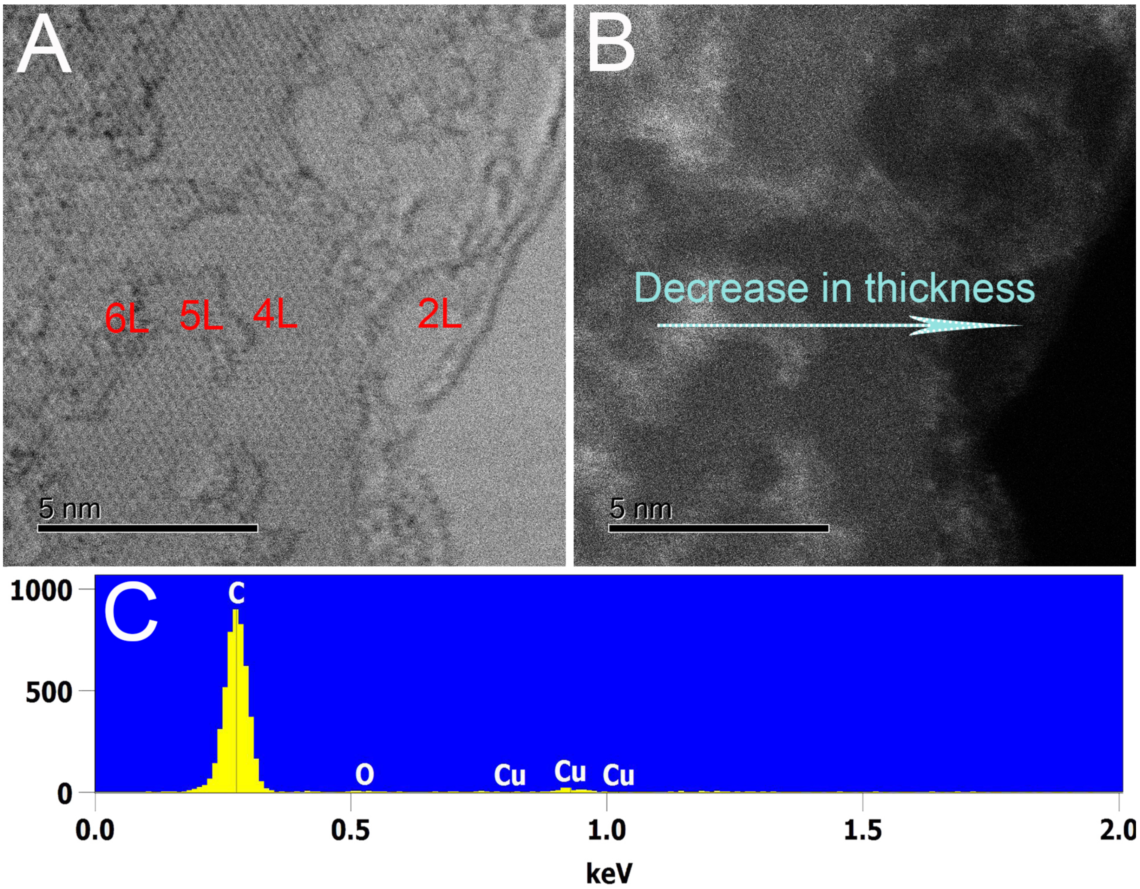

Supplementary Figure 8. A) Bright field and B) HAADF images of solvent-exfoliated graphene from graphite (99.9% purity). The brighter areas in the HAADF image are not indicative of silicon-based contamination but are due to regions of thicker multi-layer graphene sheets, since the HAADF image contrast is a function of both Z^2 and thickness. Side by side comparison of the BF and HAADF images confirms the correlation between brighter areas and greater number of layers. The number of layers is marked on the BF image (A). C) The EDS spectrum shows only a carbon peak and very little oxygen and no silicon.

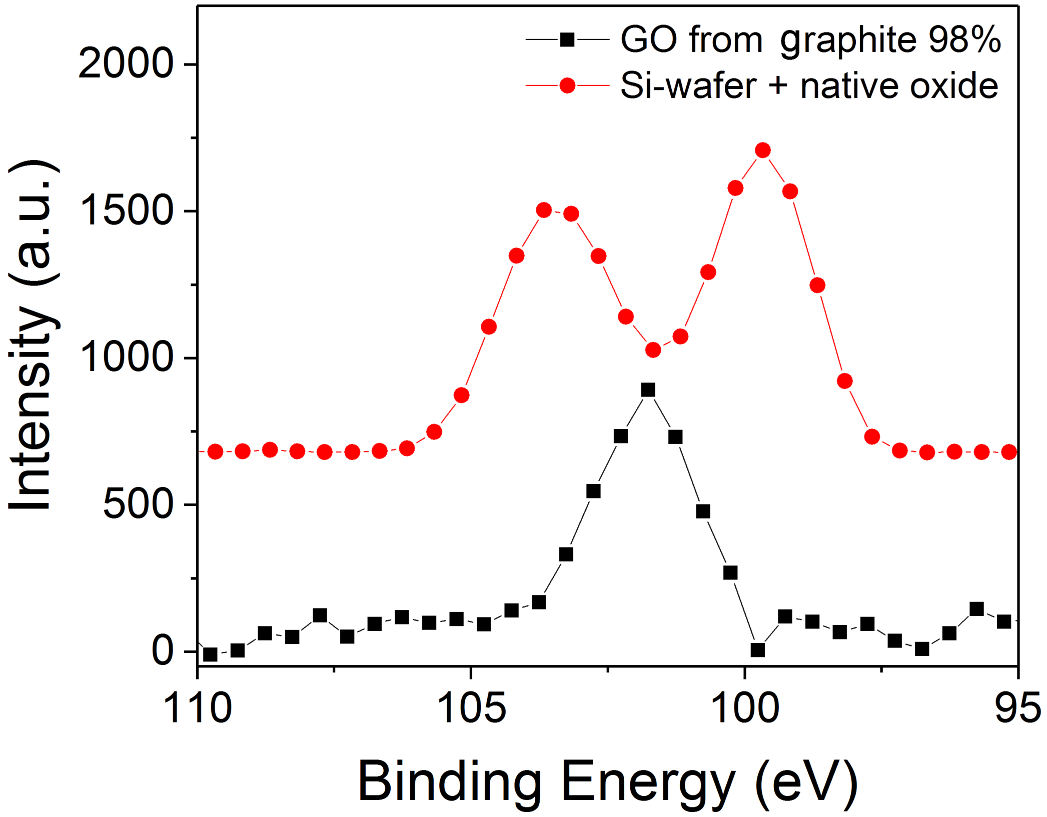

Supplementary Figure 9. Comparison of the XPS Si 2p spectral region of contaminated GO film with that from a bare SiO_2 substrate.

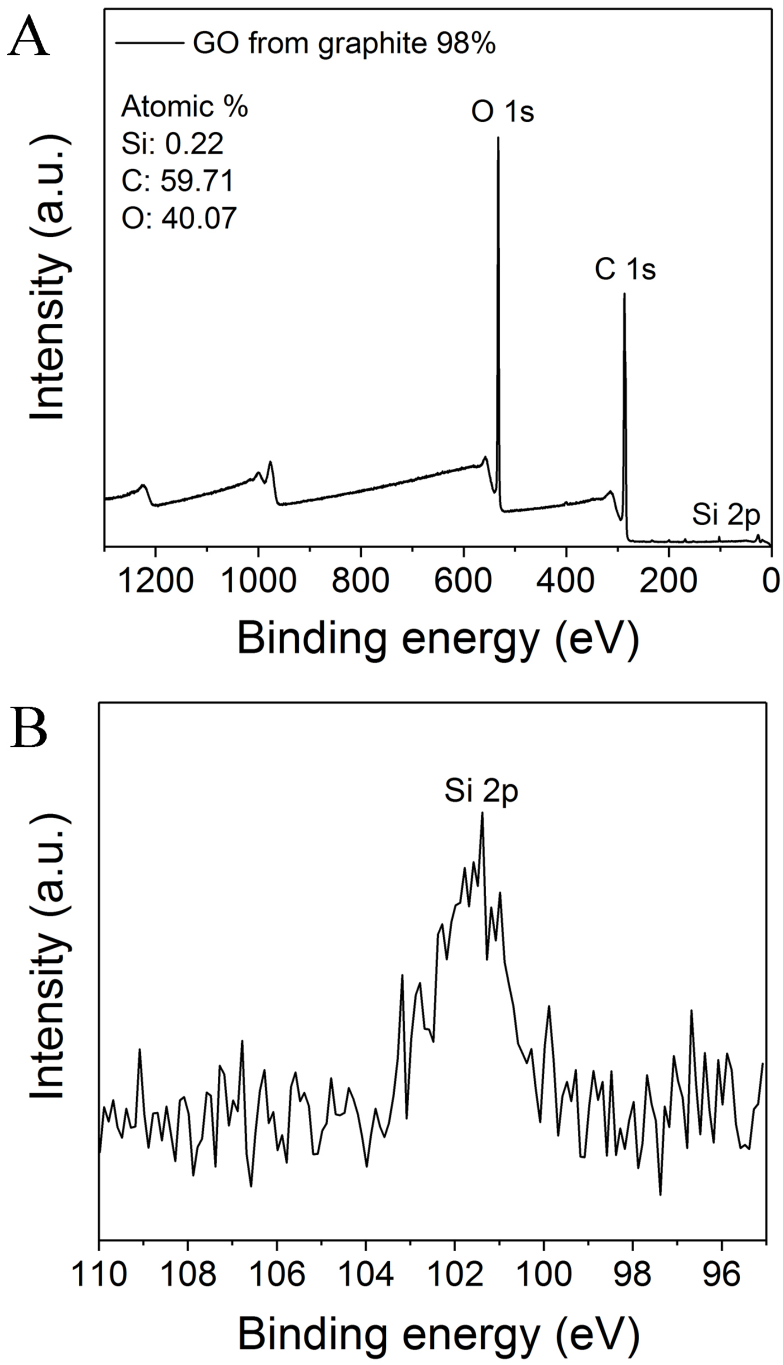

Supplementary Figure 10. XPS survey of an extensively washed GO from low purity graphite (98%). Please note the observed elements are Si (0.22 atomic %), C (59.71 atomic %) and O (40.07 atomic %). We should point that out we have observed traces of Cl and S in non-extenively washed samples (residue from H_2SO_4 treatment and HCl washing process, respectively). Cl and S were removed effectively by further washing and purification. However, the Si impurity was not removed over the purification process.

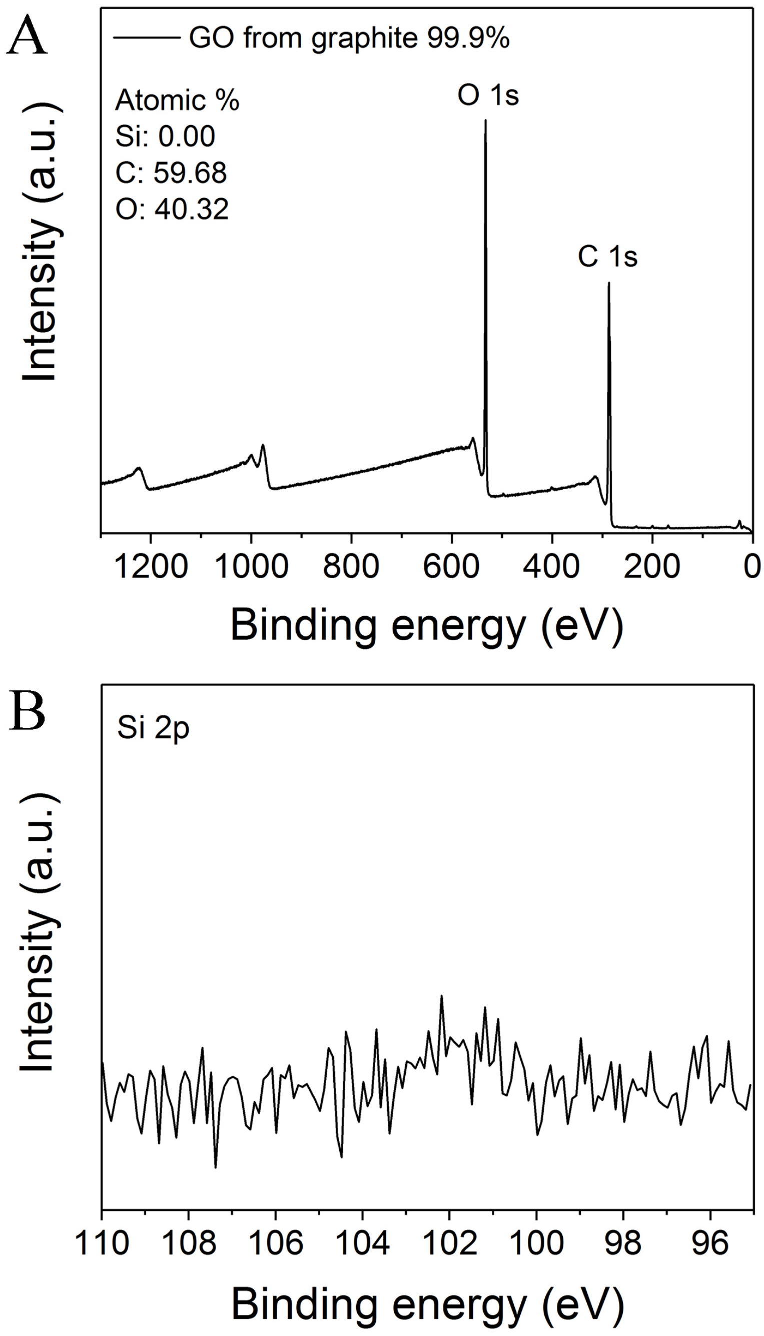

Supplementary Figure 11. XPS survey of an extensively washed GO from high purity graphite (99.9%). Si (0.00 atomic %), C (59.68 atomic %) and O (40.32 atomic %). Please note, the Si surface impurity was not found in the high purity sample as it was handled very carefully and was kept only under ultra-high turbo vacuum.

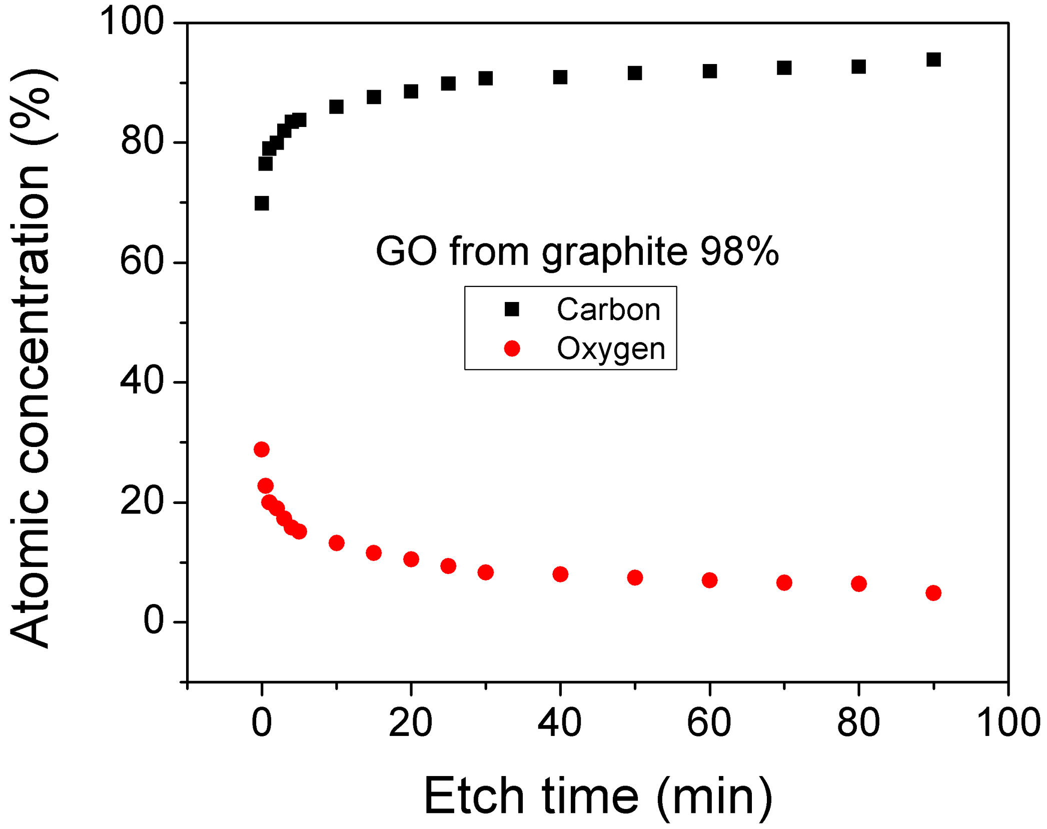

Supplementary Figure 12. Atomic concentrations of carbon and oxygen in GO derived from graphite (98% purity) as function of etching time (determined by XPS).

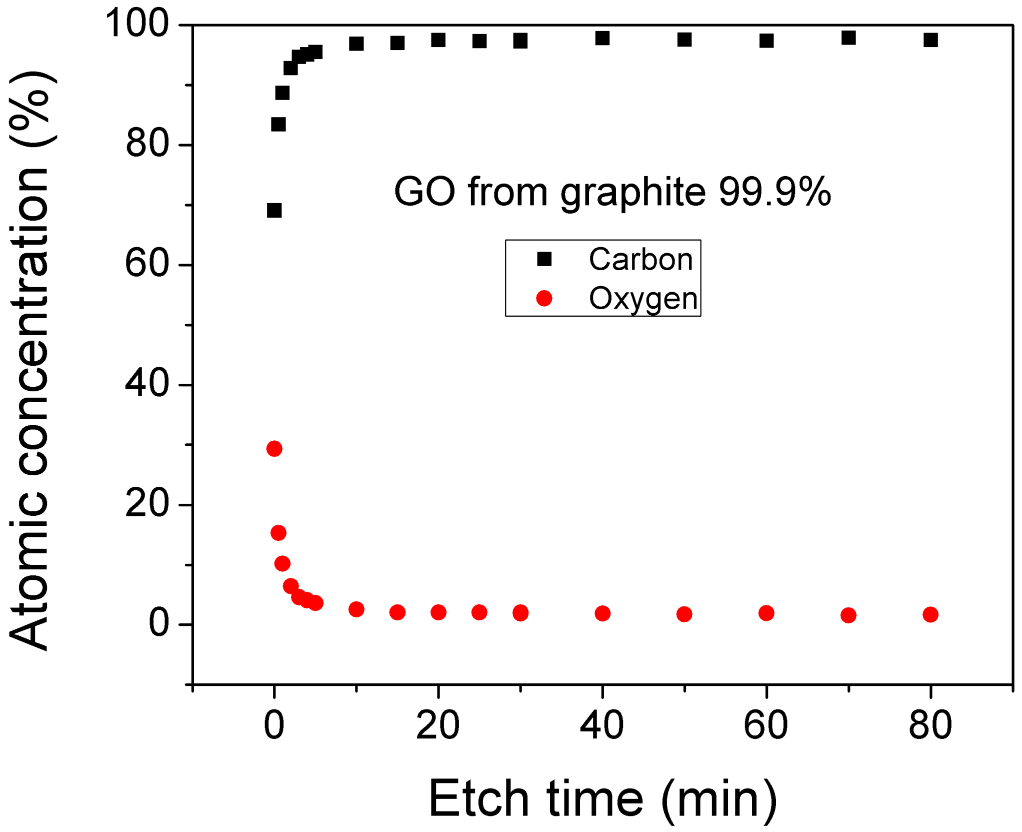

Supplementary Figure 13. Atomic concentrations of carbon and oxygen in GO derived from graphite (99.9% purity) as function of etching time (determined by XPS).

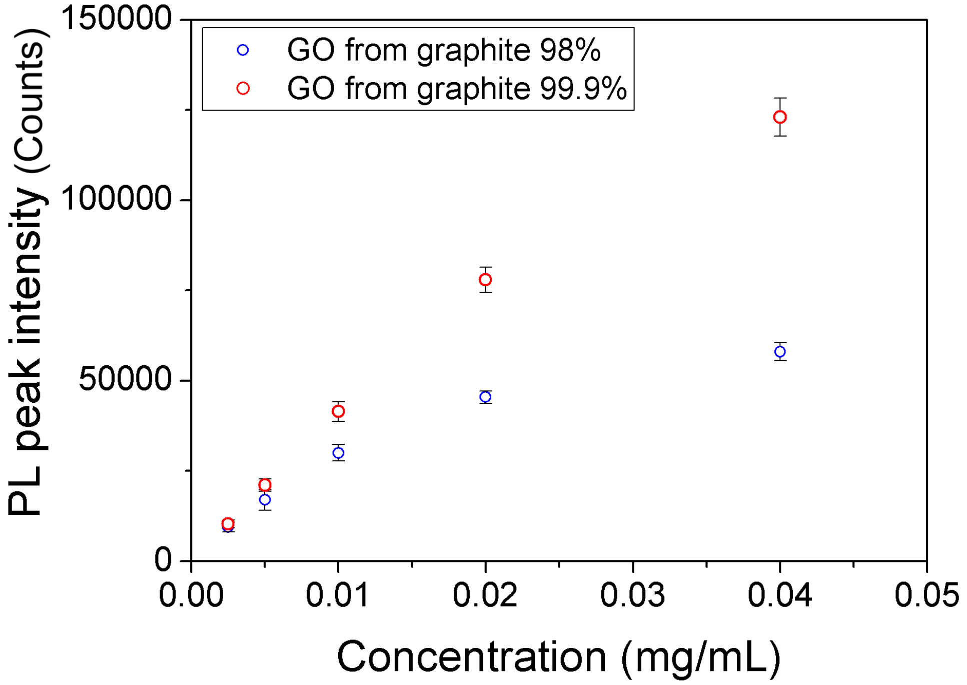

Supplementary Figure 14. Comparisons of the photo-luminescence (PL) (λ_{ex} = 350 nm) peak intensity of GO solution as function of GO concentration. Much lower emission from the GO synthesised from lower purity graphite (98%) is found compared to the purer GO (99.9%). This is due to silicon-based contamination on the surface of GO. All three kinds of functional groups (CO, C=O and O=C-OH), which are involved in the fluorescence of GO, are disrupted by the silicon-based impurity. Moreover, this impurity can act as a barrier on the surface that adversely affects the emission.

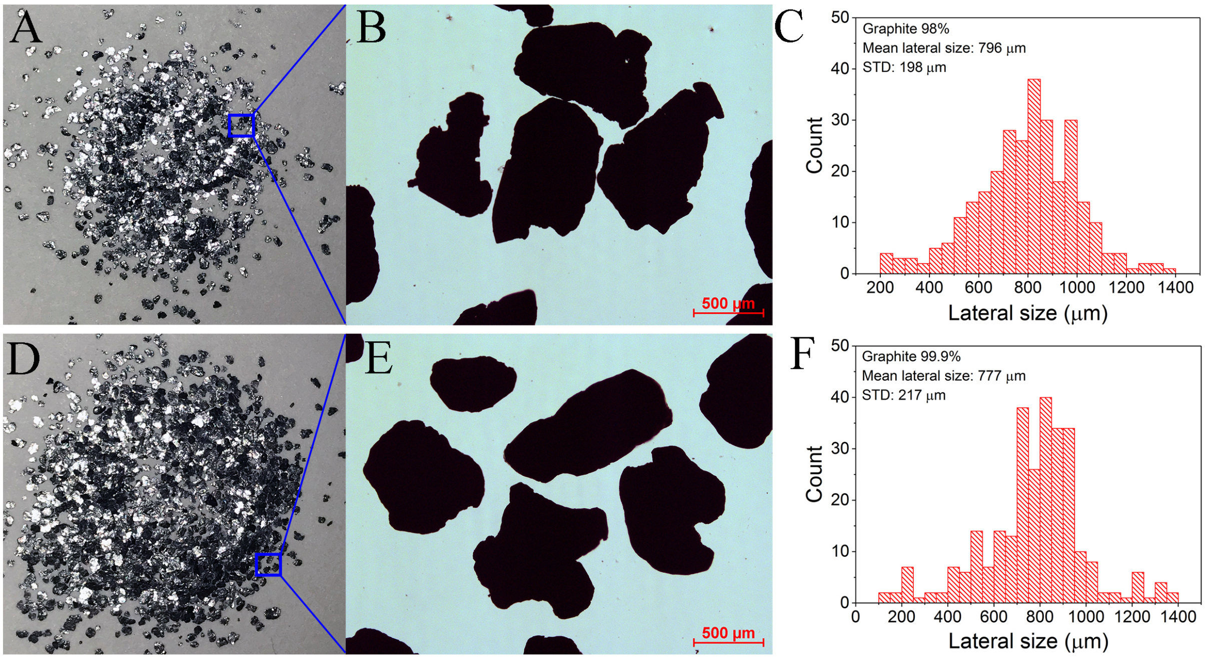

Supplementary Figure 15. Representative micrographs of graphite flakes along with flake size distribution of (A-C) low purity graphite (98%) and (B-F) high purity graphite (99.9%) used in this study to synthesise GO for the device fabrication. Graphite flakes with similar mesh (-20+80) were used to assure synthesis of GO with analogous sheet sizes distribution (Figure S16). The lateral size of each graphite flake was determined from an optical microscopy image and analyzed using image analysis software (imageJ, http://rsb.info.nih.gov/ij/). The lateral size of the graphite flake was defined as the diameter of an equal-area circle. Please note, these two graphites contained very similar grain size distributions, which resulted in similar sheet size distribution in the resultant GO (Figure S16). T-test confirmed there was no difference between the mean lateral sizes of both graphite samples.

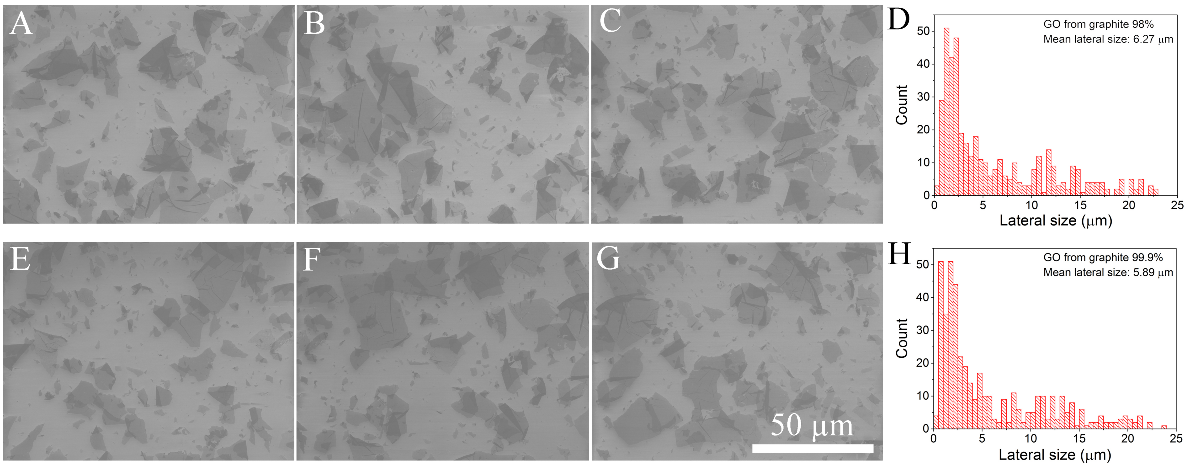

Supplementary Figure 16. SEM micrographs along with the lateral size distribution comparing GO sheets synthesised from (A-D) low purity (98%) and (E-H) high purity (99.9%) graphite. The lateral size distributions of isolated GO sheets were determined from the SEM images and analyzed using image analysis software (imageJ). The lateral size of the GO sheets was defined as the diameter of an equal-area circle. T-test confirmed there was no difference between the mean lateral sizes of both GO samples.

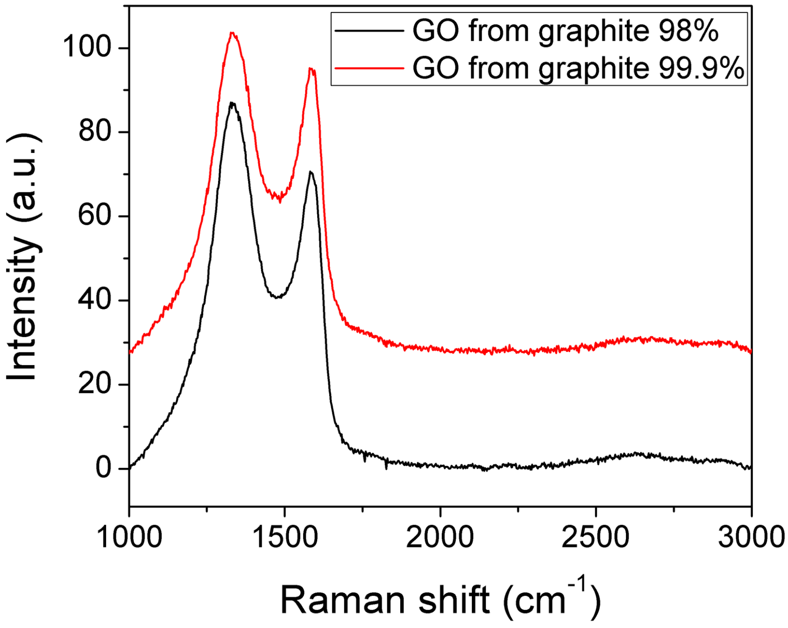

Supplementary Figure 17. Raman spectra of GO films synthesised from low purity (98%) and high purity (99.9%) graphite. The plots are offset vertically to increase the legibility.

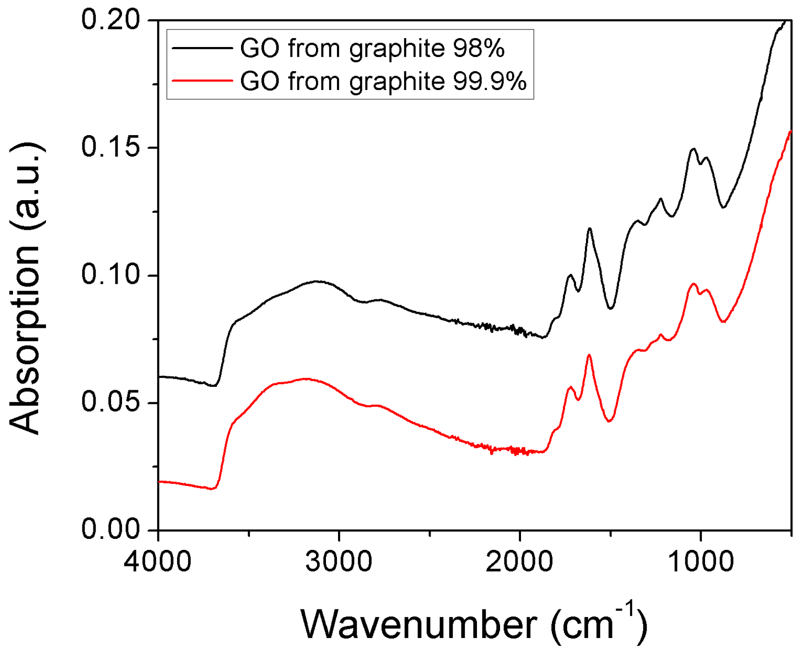

Supplementary Figure 18. FTIR spectra of GO films synthesised from graphite of varying purities. The plots are offset vertically to increase the legibility.

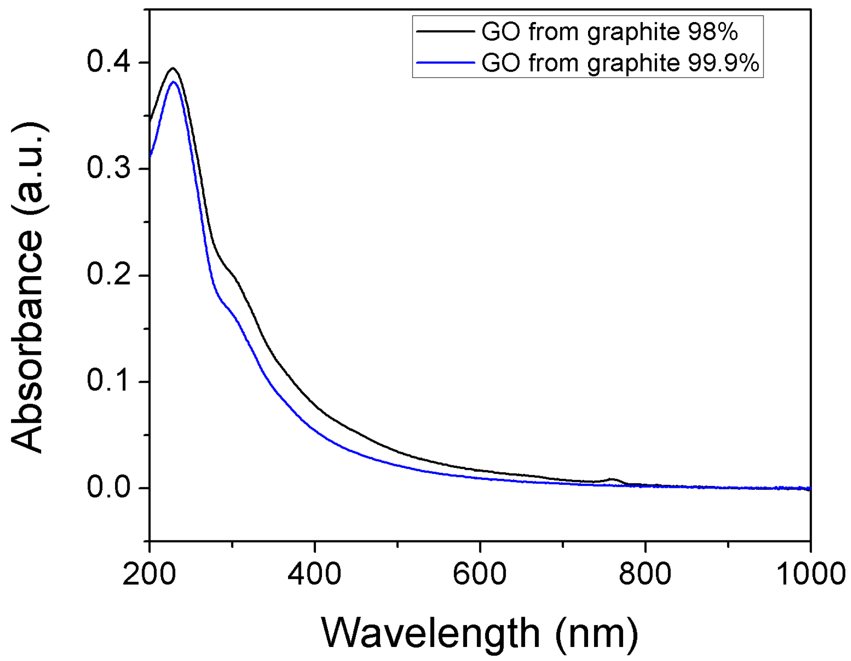

Supplementary Figure 19. UV-Vis spectra of GO solution synthesised from low purity (98%) and high purity (99.9%) graphite at 0.1 mg/ml concentration.

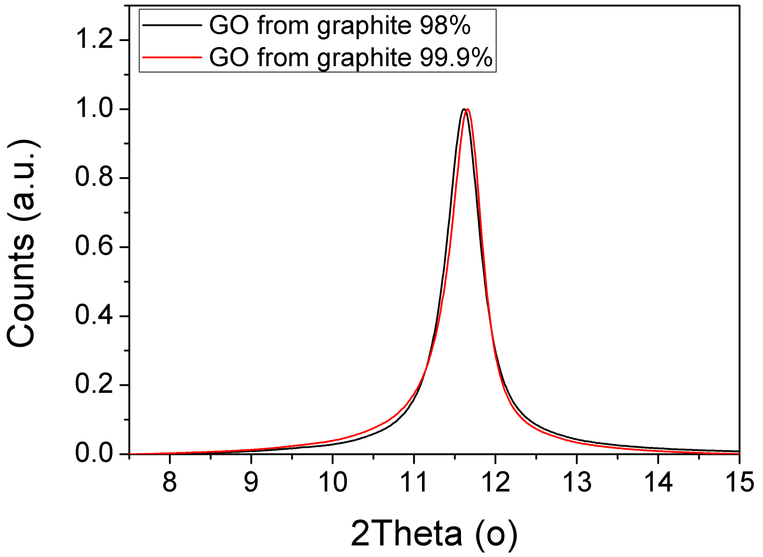

Supplementary Figure 20. X-ray diffraction patterns of GO films synthesised from low purity (98%) and high purity (99.9%) graphite. Please note, the position of the peak at 2θ of ~11.5^{o} shifted very slightly toward the lower 2θ (higher *d-spacing*) in GO from low purity graphite (98%). In order to verify whether this shift is real or not, the instrument was calibrated carefully using NaCl crystals. Here, the measurements were performed using an auto-sampler unit and then without changing the samples or getting them out, the NaCl crystals were sputtered on top of the samples and check for any deviations in the peak position, if there is any.

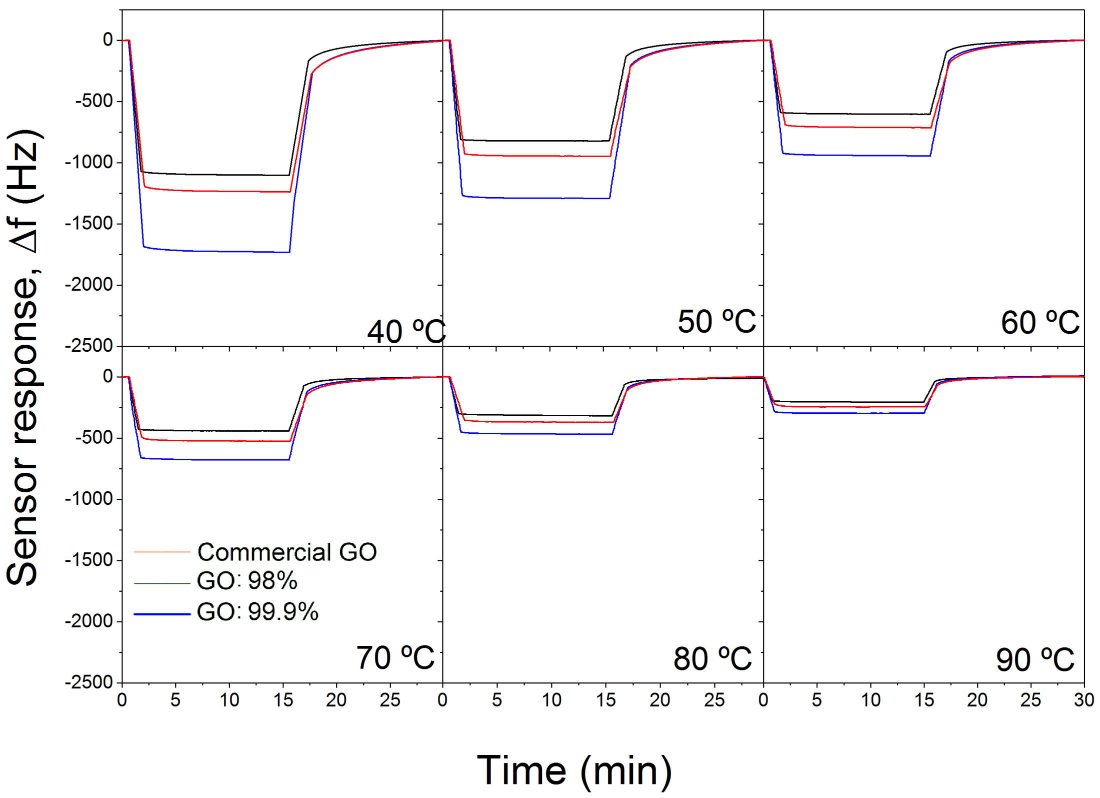

Supplementary Figure 21. Sensor response profiles at different operating temperatures. The influence of temperature on humidity sensor response was tested using 50 %RH @ 27°C, while the operating temperature ranged from 27 to 90 °C with an increment of 10 °C. In order to study the influence of temperature on humidity, two different factors need to be considered. Firstly, QCM device, being made of AT-cut quartz is most stable at room temperature and is shown previously to be stable up to 105°C due to its temperature coefficient[26], however it would produce lower signal to noise ratio as the operating temperature increases[17]. The second factor comes from the interaction between the analyte and the sensitive layer. The combination of these two factors could result in either increase or decrease in response and S/N with increasing temperature. Here, the decreasing response behaviour is thought to be due to reduced interaction of the H_2O molecules with the surface as a result of the higher vapour pressure of H_2O at higher temperatures.

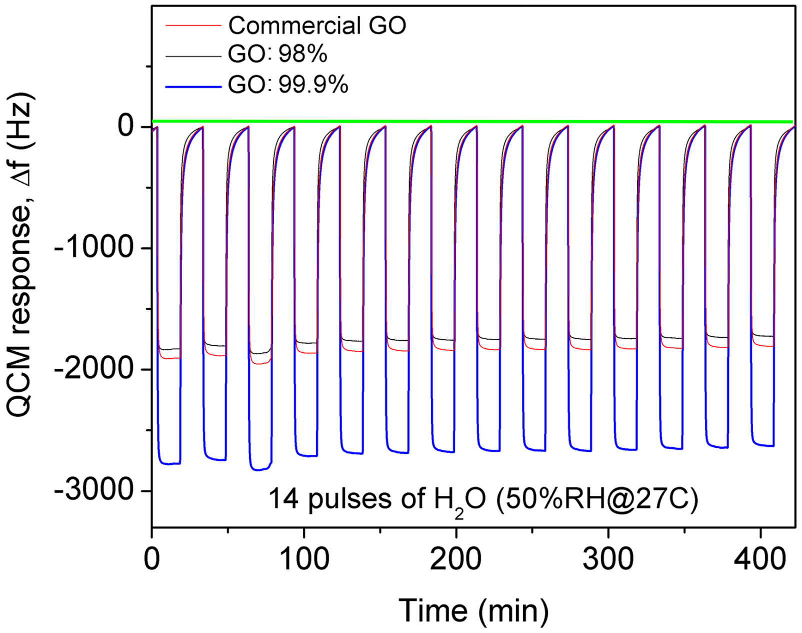

Supplementary Figure 22. Repeatability test consisting of 14 pulses of humidity with 15 min on/off sequences tested against 50 % RH.

Supplementary References:

1 Ohira, S.-I., Goto, K., Toda, K. & Dasgupta, P. K. A Capacitance Sensor for Water: Trace Moisture Measurement in Gases and Organic Solvents. *Analytical Chemistry* **84**, 8891-8897,(2012).
2 Gan, X. *et al.* High performance graphene oxide-based humidity sensor integrated on a photonic crystal cavity. *Applied Physics Letters* **110**, 151107,(2017).
3 Borini, S. *et al.* Ultrafast Graphene Oxide Humidity Sensors. *ACS Nano* **7**, 11166-11173,(2013).
4 Sabri, Y. M., Kandjani, A. E., Ippolito, S. J. & Bhargava, S. K. Ordered Monolayer Gold Nano-urchin Structures and Their Size Induced Control for High Gas Sensing Performance. *Scientific Reports* **6**, 24625,(2016).
5 Zhao, X., Long, Y., Yang, T., Li, J. & Zhu, H. Simultaneous High Sensitivity Sensing of Temperature and Humidity with Graphene Woven Fabrics. *ACS Applied Materials & Interfaces* **9**, 30171-30176,(2017).
6 Bi, H. *et al.* Ultrahigh humidity sensitivity of graphene oxide. *Scientific Reports* **3**, 2714,(2013).
7 Zhang, K.-L., Hou, Z.-L., Zhang, B.-X. & Zhao, Q.-L. Highly sensitive humidity sensor based on graphene oxide foam. *Applied Physics Letters* **111**, 153101,(2017).
8 Cheon, K. H., Cho, J., Kim, Y.-H. & Chung, D. S. Thin Film Transistor Gas Sensors Incorporating High-Mobility Diketopyrrolopyrole-Based Polymeric Semiconductor Doped with Graphene Oxide. *ACS Applied Materials & Interfaces* **7**, 14004-14010,(2015).
9 Phan, D.-T., Park, I., Park, A.-R., Park, C.-M. & Jeon, K.-J. Black P/graphene hybrid: A fast response humidity sensor with good reversibility and stability. *Scientific Reports* **7**, 10561,(2017).
10 Li, N., Chen, X., Chen, X., Ding, X. & Zhao, X. Ultrahigh humidity sensitivity of graphene oxide combined with Ag nanoparticles. *RSC Advances* **7**, 45988-45996,(2017).
11 Hwang, S.-H., Kang, D., Ruoff, R. S., Shin, H. S. & Park, Y.-B. Poly(vinyl alcohol) Reinforced and Toughened with Poly(dopamine)-Treated Graphene Oxide, and Its Use for Humidity Sensing. *ACS Nano* **8**, 6739-6747,(2014).
12 Yasaei, P. *et al.* Stable and Selective Humidity Sensing Using Stacked Black Phosphorus Flakes. *ACS Nano* **9**, 9898-9905,(2015).
13 Sumeet, W. *et al.* Defining the role of humidity in the ambient degradation of few-layer black phosphorus. *2D Materials* **4**, 015025,(2017).
14 Muckley, E. S. *et al.* Multimodality of Structural, Electrical, and Gravimetric Responses of Intercalated MXenes to Water. *ACS Nano* **11**, 11118-11126,(2017).
15 Yan, H., Zhang, L., Yu, P. & Mao, L. Sensitive and Fast Humidity Sensor Based on A Redox Conducting Supramolecular Ionic Material for Respiration Monitoring. *Analytical Chemistry* **89**, 996-1001,(2017).
16 Wu, S. *et al.* Organic Field-Effect Transistors with Macroporous Semiconductor Films as High-Performance Humidity Sensors. *ACS Applied Materials & Interfaces* **9**, 14974-14982,(2017).
17 Kabir, K. M. M. *et al.* Mercury Sorption and Desorption on Gold: A Comparative Analysis of Surface Acoustic Wave and Quartz Crystal Microbalance-Based Sensors. *Langmuir* **31**, 8519-8529,(2015).

18 Zhou, Z. *et al.* Antiferromagnetic Copper(II) Metal–Organic Framework Based Quartz Crystal Microbalance Sensor for Humidity. *Crystal Growth & Design* **17**, 6719-6724,(2017).
19 Liu, R., Gong, T., Zhang, K. & Lee, C. Graphene oxide papers with high water adsorption capacity for air dehumidification. *Scientific Reports* **7**, 9761,(2017).
20 Chen, X., Zhang, J., Wang, Z., Yan, Q. & Hui, S. Humidity sensing behavior of silicon nanowires with hexamethyldisilazane modification. *Sensors and Actuators B: Chemical* **156**, 631-636,(2011).
21 Yao, Y., Chen, X., Guo, H., Wu, Z. & Li, X. Humidity sensing behaviors of graphene oxide-silicon bi-layer flexible structure. *Sensors and Actuators B: Chemical* **161**, 1053-1058,(2012).
22 Kuang, Q., Lao, C., Wang, Z. L., Xie, Z. & Zheng, L. High-Sensitivity Humidity Sensor Based on a Single SnO2 Nanowire. *Journal of the American Chemical Society* **129**, 6070-6071,(2007).
23 Zhao, J. *et al.* Highly Sensitive MoS2 Humidity Sensors Array for Noncontact Sensation. *Advanced Materials* **29**, 1702076,(2017).
24 Sabri, Y. M. *et al.* Gold nanospikes based microsensor as a highly accurate mercury emission monitoring system. *Scientific Reports* **4**, 6741,(2014).
25 Lobo, D. E., Fu, J., Gengenbach, T. & Majumder, M. Localized Deoxygenation and Direct Patterning of Graphene Oxide Films by Focused Ion Beams. *Langmuir* **28**, 14815-14821,(2012).
26 Takashi, Y., Keigo, I., Shigeo, K. & Michiaki, T. Temperature Stability of Surface Acoustic Wave Resonators on In-Plane Rotated 33° Y-Cut Quartz. *Japanese Journal of Applied Physics* **42**, 3136,(2003).
